# Chemical Genomic-Based Pathway Analyses for Epidermal Growth Factor-Mediated Signaling in Migrating Cancer Cells

**DOI:** 10.1371/journal.pone.0096776

**Published:** 2014-05-12

**Authors:** Shigeyuki Magi, Yuya Saeki, Masato Kasamatsu, Etsu Tashiro, Masaya Imoto

**Affiliations:** Department of Biosciences and Informatics, Faculty of Science and Technology, Keio University, Yokohama, Kanagawa Japan; Hungarian Academy of Sciences, Hungary

## Abstract

To explore the diversity and consistency of the signaling pathways that regulate tumor cell migration, we chose three human tumor cell lines that migrated after treatment with EGF. We then quantified the effect of fifteen inhibitors on the levels of expression or the phosphorylation levels of nine proteins that were induced by EGF stimulation in each of these cell lines. Based on the data obtained in this study and chemical-biological assumptions, we deduced cell migration pathways in each tumor cell line, and then compared them. As a result, we found that both the MEK/ERK and JNK/c-Jun pathways were activated in all three migrating cell lines. Moreover, GSK-3 and p38 were found to regulate PI3K/Akt pathway in only EC109 cells, and JNK was found to crosstalk with p38 and Fos related pathway in only TT cells. Taken together, our analytical system could easily distinguish between the common and cell type-specific pathways responsible for tumor cell migration.

## Introduction

Cell migration is central to many physiological processes, including embryonic development, wound repair, immune responses, as well as tumor cell invasion and metastasis [1]. When a tumor cell moves, several signaling pathways are initiated through receptor tyrosine kinases (RTKs), G protein-coupled receptors (GPCRs), integrins, and other receptors. A notable example of a RTK is the epidermal growth factor receptor (EGFR), which is activated by binding of its ligand, epidermal growth factor (EGF) [2]. The activation of EGFR leads to the activation of one or more intermediate signaling network branches which regulate cell motility, such as the extracellular-regulated kinase (ERK) pathway [3], the phosphoinositide 3-OH kinase (PI3K) pathway [4], the Janus kinase (Jak) pathway [5], the c-Jun NH2 terminal kinase (JNK) pathway, and the p38 pathway [6], [7].

The core elements of the intracellular migration-signaling network have been demonstrated in previous studies. However, it is likely that the signaling molecules that regulate cell migration in one cancer cell may not regulate cell migration in other genetically distinct cancer cells. Several previous reports have indicated that each type of cancer cell initiates migration in different contexts using distinct molecular repertoires, even though the same basic process of cell migration is induced [8], [9]. Therefore, understanding the diversity and generality of signaling pathways that regulate tumor cell migration in various cell types is important not only for basic research into cell migration, but also for the development of anti-metastatic anti-tumor drugs.

To address this issue, we previously investigated the effect of small molecule inhibitors on ten cell migration system types. We distinguished between the common and cell type-specific signals responsible for cell migration [10]. Previous research has indicated which molecules are actually involved in the cell migration of each cancer cell type. However, the signaling networks of these molecules that regulate cell migration remain unclear. In this report, to address this issue, we utilized an approach combining chemical genetics and systems biology, which has gradually been recognized as a useful method for deducing signaling pathway networks [11]. In our previous report, we found that three cancer cell lines (i.e., epidermal carcinoma A431 cells, esophageal carcinoma EC109 cells, and thyroid carcinoma TT cells) acquired cell motility by EGF stimulation, but chemosensitivity cluster analysis showed that A431 cells and EC109 cells are clustered into the same cluster, on the other hand, TT cells are classified into the different cluster. Therefore, in this study, to reveal the diversity and commonality of EGF-induced signaling pathway regulating cell migration in these three cells, we quantitatively examined the effect of chemical inhibitors on EGF-induced expression levels or the phosphorylation level of several signaling molecules to identify which signaling molecule acts upstream of other signaling molecules. Using the results of these experiments, we mapped a cell migration pathway in each cancer cell line, and compared the pathway maps to reveal the network topology as being either common to all cancer cells or specific to certain cell types.

## Results

### The different activation patterns of EGF signaling among three cancer cell lines

**Figure 1:**
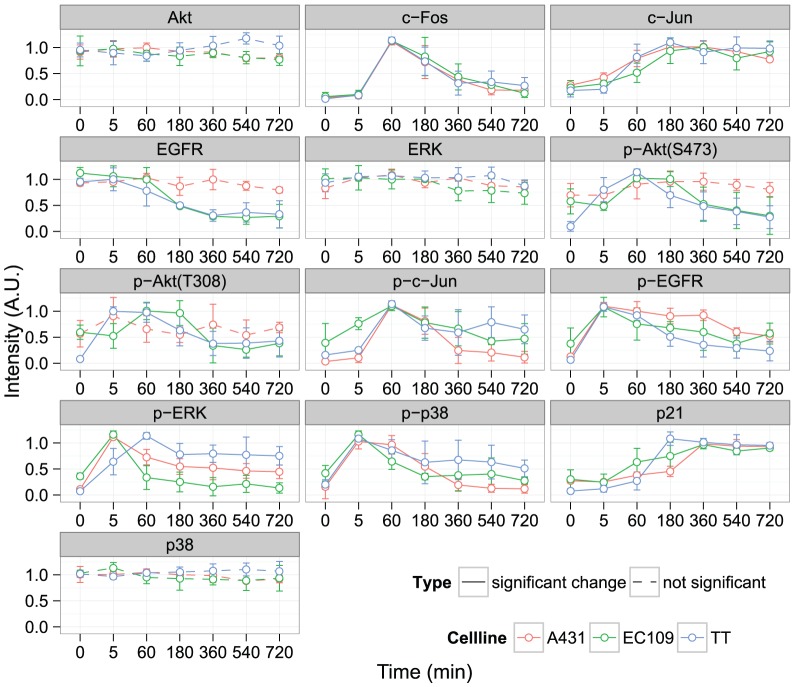
EGF-induced time course of protein phosphorylation and expression in three cancer cell lines. A431 cells, EC109 cells, and TT cells were stimulated by EGF (30 ng ml^−1^) for the indicated time and total cell lysates were subjected to western blotting. A.U., arbitrary unit normalized by the intensity of actin. The means and SDs of three independent experiments are shown. The graph colors represent each cell line data: A431 cells (red), EC109 cells (green), and TT cells (blue). The dashed line indicates that EGF stimulation did not cause a significant change of A.U. of each molecule (One-way ANOVA). Original immunoblot images are shown in **Figure S1**.

Firstly, we detected the phosphorylation or expression of signaling molecules induced by EGF in three cancer cell lines over a time course (**Figure 1** and **S1**). Autophosphorylation of the EGF receptor and subsequent EGF-induced phosphorylation of p38 were both observed in all cell lines after 5 min following EGF stimulation, as is well known. The increase in the expression of c-Fos and the phosphorylation of c-Jun were observed in all cell lines 1 h after EGF stimulation. On the other hand, several other molecules showed different time-dependent activation profiles between the three cancer cell lines. For example, the phosphorylation of Akt (p-Akt, S473 and T308 residues) was induced between 5 min and 1 h following EGF stimulation in EC109 cells and TT cells. However, the phosphorylation levels of p-Akt (S473) and p-Akt (T308) in A431 cells were somewhat constant even after EGF stimulation of up to 12 h. In addition, the relative intensity of p-Akt (T308) showed a maximum intensity 5 min after EGF stimulation in TT cells, but after 1 h in EC109 cells. The pattern of ERK phosphorylation (p-ERK) is also different among the three cancer cell lines. ERK was transiently phosphorylated by EGF stimulation in all three cells, whereas its peak was observed 5 min after EGF stimulation in A431 cells and EC109 cells, and after 1 h in TT cells. The expression level of EGFR began to decrease following EGF stimulation in EC109 cells and TT cells, but not in A431 cells.

### The effects of migration inhibitors on EGF-induced migration signaling

**Figure 2:**
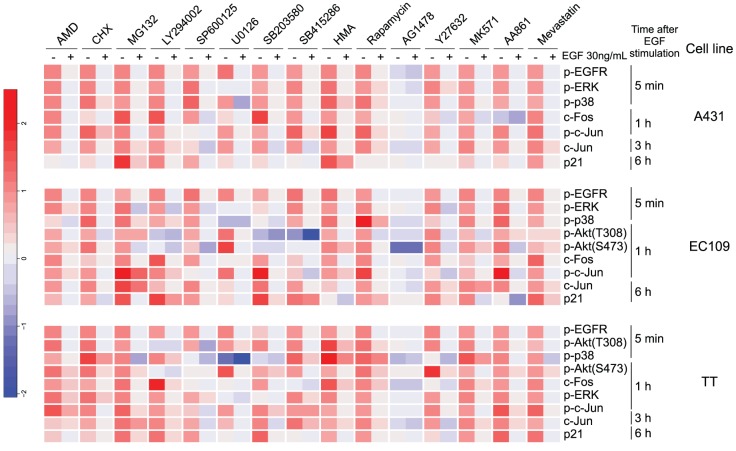
Effects of small molecule inhibitors on EGF-induced migration signaling in three cancer cell lines. Heat maps depict the effects of small molecule inhibitors on EGF-induced signaling in A431 cells (top), EC109 cells (middle), and TT cells (bottom). Data were normalized by centering non-treat condition as 0, and scaling EGF-treated condition to 1. All cell lines were treated with EGF after pre-treatment with inhibitors for 15 min. After the indicated time, the cells were collected and subjected to western blotting. AMD; Actinomycin D, CHX; Cycloheximide, HMA; Herbimycin A. The concentration of chemical inhibitors are listed in Table S2. Original immunoblot images are shown in **Figure S2**.

Next, we examined the effects of 15 inhibitors, which affected the ability of cells to migrate in our previous report [10], on EGF-induced phosphorylation or expression of these signaling molecules at the time point showing the highest intensity of each signaling molecule as indicated by time course data (**Table S1**) in each cancer cell line (**Figure S2**). **Table S2** lists the names and the experimental concentrations used of the chemical inhibitors of signal transduction used in this study, and their modes of action. The immunoblot images in **Figure S2** include bands for each of four conditions: negative control (EGF - and inhibitor -, lane 1), positive control (EGF + and inhibitor -, lane 2), both EGF and inhibitor-treated condition (EGF + and inhibitor +, lane 3), and inhibitor-treated condition (EGF - and inhibitor +, lane 4). To quantify the effect of the inhibitors, we then normalized the signal intensity so that lane 1 (i.e., non-treated condition) was designated as 0, and lane 2 (i.e., the EGF-treated condition) was designated as 1. Based on these standards, we quantified the relative signal intensity of lanes 3 and 4, and finally we obtained quantitative dataset of the effect of chemical inhibitors on EGF-induced migration signaling in each cell line (**Figure 2**).

### Deduction of migration signaling in three cancer cell lines

**Figure 3:**
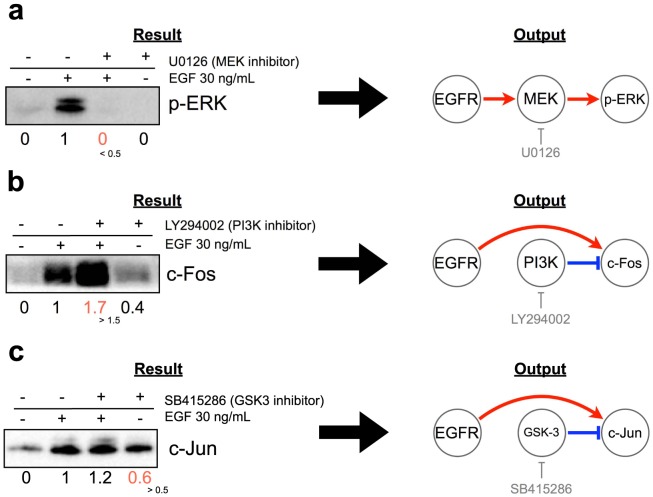
The concept for deduction of signaling pathway. Detailed description is presented in the Results section and Experimental Procedures section.

**Figure 4:**
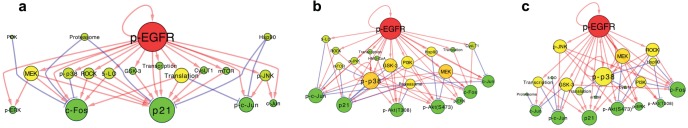
EGF-induced migration signaling network in three cancer cell lines. EGF-induced migration signaling network in (**a**) A431 cells, (**b**) EC109 cells, and (**c**) TT cells. The threshold was set at ±50% from positive control. The edge color is decided based on the sign of edges; red indicates positive signaling, and blue indicates negative signaling. The size of the node indicates the number of connecting edges. The color of the node indicates the number of output pathways: a gradient color scale from green to red, interpolated over yellow.

We then attempted to deduce the signaling network regulating cell migration in each cancer cell line based on the chemosensitivity profiles obtained on the expression status of EGF-induced signaling molecules. In the field of chemical biology, a signaling pathway may be deduced using the concept described in **Figure 3** (see also, Experimental Procedures). To determine whether inhibitors perturbed the signaling molecules or not, we defined ±0.5 as a threshold. In the case of **Figure 3a**, the MEK inhibitor U0126 suppressed the EGF-induced phosphorylation of ERK (p-ERK) by more than 50%. In such cases, we defined two positive signal regulations; one from EGFR to MEK, and the other from MEK to p-ERK. In the case of **Figure 3b**, the PI3K inhibitor LY294002 increased EGF-induced c-Fos expression by more than 50%. In these cases, we defined the existence of one positive regulation relationship from EGFR to c-Fos and one negative regulation relationship from PI3K to c-Fos. In the case of **Figure 3c**, the GSK-3 inhibitor SB415286 increased c-Jun expression without EGF stimulation. In these cases, we defined the existence of one positive regulation relationship from EGFR to c-Jun and one negative regulation relationship from GSK-3 to c-Jun. In this way, based on the semi-quantitative profile (**Figure 2** and the concept described in **Figure 3**), we deduced a signaling network for each cancer cell line as a signed directed graph (**Figure 4**). The EGF-induced migration pathway in A431 cells consisted of 19 nodes and 39 edges (**Figure 4a**), the pathway in EC109 cells consisted of 22 nodes and 62 edges (**Figure 4b**), and the pathway in TT cells consisted of 21 nodes and 51 edges (**Figure 4c**). The reason why the number of pathways in A431 cells is fewer than in the other two cell lines might be that levels of p-Akt(T308) and p-Akt(S473) could not be evaluated in A431 cells.

### Comparison of signaling pathways in each cancer cell line

**Figure 5:**
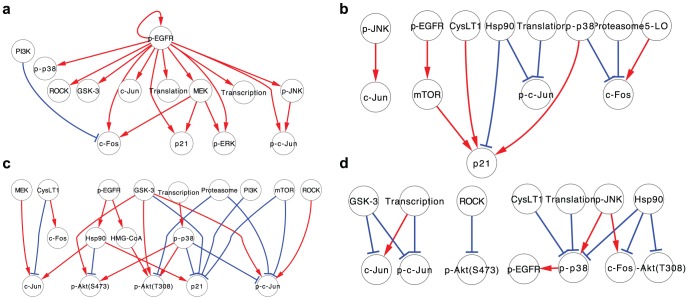
Comparison of signaling network among three cancer cell lines. (**a**) Common EGF-induced signaling network among three cancer cell lines. (**b∼d**) Specific EGF-induced signaling network in (**b**) A431 cells, (**c**) EC109 cells, and (**d**) TT cells. The edge color is decided based on the sign of edges; red indicates positive signaling, and blue indicates negative signaling.

Based on these pathway maps, we extracted the common structure of signaling pathways for cell migration in all three cancer cell lines examined in this study. **Figure 5a** represents the common topology among the three cell lines. This common pathway includes the MEK/ERK/c-Fos pathway and the JNK/c-Jun pathway, which have both been widely researched in the field of cell migration [6], [7], [12], [13]. We also found that MEK regulated expression levels of c-Fos and p21, and that PI3K suppressed the expression levels of c-Fos in the three cancer cell lines. On the other hand, other EGFR-regulated signals such as ROCK and CysLT1 did not have any output pathways, suggesting that the downstream signals of these molecules may vary between each cancer cell line. Next, we evaluated the specific structures of the migration-related signaling pathways in each of the cancer cell lines (**Figure 5b** **∼d**). The specific pathway in A431 cells includes only 11 pathways, including p-JNK→c-Jun expression and 5-lipoxygenase (5-LO)→c-Fos. The number of specific pathways in EC109 cells and in TT cells are 24 and 13, respectively, indicating that about one-fifth to one-third of pathways in these cells are cell-type-dependent pathways.

**Figure 6:**
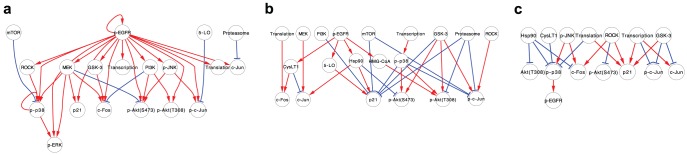
Comparison of signaling network between EC109 cells and TT cells. (**a**) Common signaling network between EC109 cells and TT cells. (**b**) Specific EGF-induced signaling network in EC109 cells. (**c**) Specific EGF-induced signaling network in TT cells. The edge color is decided based on the sign of edges; red indicates positive signaling, and blue indicates negative signaling.

In a previous study, we predicted that similar pathways induce and regulate cell migration in A431 cells and EC109 cells, but these pathways may be different from TT cells [10]. To clarify the difference in signaling pathways between the two groups, we then compared the signaling pathways of EC109 cells and TT cells which includes all the same nodes (**Figure 6**). **Figure 6a** shows the overlapped pathway topology in both cell lines. This pathway map includes PI3K→p-Akt, JNK→p-Akt(S473) and ROCK→p-p38, suggesting that these pathways play a consistent role in cell migration. **Figure 6b, c** presents the specific pathway topologies in EC109 cells and TT cells, respectively. A total of 29 pathways (47% of pathways in EC109 cells), such as MEK→c-Jun, GSK-3→p-Akt(S473) and HMG-CoA→pAkt(T308), are specific for EC109 cells, and 18 pathways (35% of pathways in TT cells), including p-JNK→p-p38, and ROCK→c-Fos, are specific for TT cells. The phosphorylation of p-Akt is up-regulated by p38, GSK-3, and HMG-CoA in EC109 cells, whereas there was no specific positive pathway to p-Akt in TT cells. On the other hand, JNK is required for increase of both c-Fos expression and phosphorylation of p38 in TT cells, whereas such regulation was not observed in EC109 cells. Moreover, there is an EGFR-p38 positive feedback loop in TT cells but there is no such pathway in EC109 cells. These results indicate that activation of Akt may be essential for cell migration of EC109 cells, but Akt activation pathway depends on cancer cell types. Moreover, stress-responsive MAPK pathway may be dominant in regulatory pathway of cell migration of TT cells.

## Discussion

In this study, we mapped the regulatory signaling pathways for cell migration in epidermal carcinoma A431 cells, esophageal carcinoma EC109 cells and thyroid carcinoma TT cells. By comparing them, we revealed the common pathways in the three cell lines and identified the cell-type-specific signaling pathways, based on a chemical genetic and systems biology approach. To accomplish this objective, we firstly compared the time-dependent activation patterns of signaling molecules in each cell line involved in EGF-induced cell migration. The activation patterns of some signaling molecules are partially different between the three cancer cell lines (**Figure 1**). For example, the activation patterns of c-Fos and p-p38 are quite similar among the three cancer cell lines tested, whereas the patterns of p-ERK and p-c-Jun are different. The relative intensities of sustained p-ERK and p-c-Jun in TT cells are higher than those in A431 cells and EC109 cells. Interestingly, this difference is consistent with previously reported classifications of the chemosensitivity profiles of these three cell lines [10], suggesting that sustained ERK and c-Jun phosphorylation are involved in the cell-type specific pathways for cell migration in TT cells. Considering a positive feedback mechanism by which sustained JNK activity can promote ERK signaling through IRS-2 [14] was reported, these mechanisms may also operate in TT cell migration. By contrast, the phosphorylation of Akt was induced by EGF stimulation in EC109 cells and TT cells, but the phosphorylation levels of Akt were somewhat constant in A431 cells following EGF stimulation. Previously, we reported that EGF-induced migration of A431 cells is suppressed by the addition of PI3K inhibitors [10]. Therefore, although EGF did not significantly induce Akt phosphorylation, steady state phosphorylated and activated Akt is required for the EGF-induced migration of A431 cells. Another difference in the time course data between the three cancer cell lines is shown by the expression level of EGFR. EGF stimulation decreased the expression level of EGFR in EC109 cells and TT cells, but not in A431 cells. When EGF binds to EGFR, EGFR is known to be rapidly internalized from the cell surface via several pathways, including clathrin-coated pits [9], [15]. Internalized receptors are either recycled to the cell surface or transported to lysosomes for degradation. It is well known that the fate of the receptors is controlled by several proteins upon internalization, such as Cbl and LRRK1 [16], [17]. Thus, our results might indicate that the regulation of EGF-induced receptor degradation is different among the three cancer cell lines, and this difference dictates the sustained activation of downstream signaling targets.

Regulatory pathways for cell migration in each cancer cell line were determined by examining the effect of cell migration inhibitors on signal transduction molecules (**Figure 4**). The overlapped pathway topology in all three tested cell lines includes JNK→p-c-Jun, which is predicted as a common regulator in a previous study. This suggests that regulatory signaling for cancer cell migration by JNK through the phosphorylation of c-Jun is actually a common mechanism in all types of cancer cells. Because we could not detect significant up-regulation of p-Akt in EGF-stimulated A431 cells in this study, EGF signaling pathway map in A431 cells could not include the regulation of p-Akt and thereby includes fewer nodes and edges than that in EC109 cells and TT cells in our analytical method. Therefore, an interpretation of the comparison among pathway maps among three cancer cell lines must be made cautiously.

To discuss and confirm the results of cluster analysis in our previous report [10], we finally compared the pathway map responsible for cell migration between EC109 cells and TT cells, and determined the overlapped or cell-type specific topology (**Figure 6**). Akt phosphorylation waves in EC109 cells are slower than that in TT cells (**Figure 1** and **Table S1**), suggesting that more intermediate proteins regulate Akt phosphorylation in EC109 cells than TT cells. In fact, the level of Akt phosphorylation was up-regulated by p38, GSK-3, and HMG-CoA in EC109 cells, while these pathways were not included in TT cells (**Figure 6b, c**). On the other hand, ERK phosphorylation waves in TT cells are slower and more continuous than in EC109 cells (**Figure 1** and **Table S1**), suggesting that more intermediate proteins regulate ERK phosphorylation in TT cells than EC109 cells. But we could not detect any specific regulation, which may sustain phosphorylation level of ERK in TT cells. We consider that another molecule we did not assess in this study may contribute to sustained ERK phosphorylation. These differences in the EGF-induced signaling pathway might reflect differences in the mode of cell migration based on cell morphology. In our previous study, we found that EC109 cells moved while retaining cell-cell contacts (collective migration), but TT cells moved as single cells (individual migration) [10]. In light of the above results, it is likely that the cancer cells move as a single cell when MAPK pathway is continuously activated, whereas, cells migrate collectively and maintain cell-cell adhesion when PI3K/Akt pathway is continuously activated by p38 and GKS-3. These distinct roles of the MAPK and PI3K pathways for migration modes are also supported by the following previous reports. MAPK activation consequently induces RhoA activation and epithelial-mesenchymal-transition, while PI3K activation suppresses RhoA activity in colon cancer [9]. In addition, PI3K is recruited to adhesion complexes [18] where their activation via cadherin signaling impacts cadherin function and strengthens cell–cell adhesion [19], [20]. This could involve PI3K-induced Rac1 activation, because E-cadherin-stimulated actin cytoskeletal reorganization requires Rac activation and PI3P-activated GEF Tiam1 [21].

Taken together, our chemical genomic based pathway analysis in three cancer cell lines demonstrate consistency and variety in the EGF-induced regulatory signaling pathway for regulating cancer cell migration between cell types using a combination approach of chemical biology and systems biology. In particular, GSK-3 and p38 regulate p-Akt in only esophageal carcinoma EC109 cells, on the other hand, JNK regulates p38 and Fos related pathway only in thyroid carcinoma TT cells. These findings indicate that some cell type-specific signaling pathway regulating cell migration would be therapeutic molecular targets for cell type specific cancer metastasis: GSK-3- and p38- related pathways in esophageal carcinoma, and stress responsive-MAPKs-relaterd pathways in thyroid carcinoma. However, to understand consistency and variety of regulatory signaling pathway more precisely, there are several issues we should deal with in the future. Although we used one inhibitor against one signaling molecule in this study, several inhibitors against one signaling molecule should be used to confirm the specificity of inhibitors. Moreover, the time when we assess the effects of inhibitors on EGF-induced phosphorylation or expression of these signaling molecules should be verified. It should be also devised how to distinguish regulatory pathway for cell migration from that for other cellular responses such as cell growth. Nevertheless, in this study, we provide a new approach for understanding the characteristics of cancer cell migration signaling, and open the potential for revealing a novel molecular pathway as a target for cancer therapy.

## Materials and Methods

### Cell Culture

A431 cells (obtained from ATCC CRL-1555) were maintained in DMEM supplemented with 5% calf serum (CS), 0.1 g l^−1^ kanamycin, 100 units ml^−1^ penicillin G, 0.6 g l^−1^ L-glutamine, and 2.5 g l^−1^ NaHCO_3_. EC109 (gifted from Dr. Doki, Osaka University, Japan), TT cells (gifted from KIRIN company, Japan) were maintained in Roswell Park Memorial Institute (RPMI) medium 1640 supplemented with 5% FBS, 0.1 g l^−1^ kanamycin, 100 units ml^−1^ penicillin G, 0.6 g l^−1^ L-glutamine, and 2.5 g l^−1^ NaHCO_3_. For routine culture, cells were incubated in a standard humidified incubator at 37°C in 5% CO_2_.

### Reagents

AG1478 (EGFR inhibitor), rapamycin (mTOR inhibitor), and Y27632 (ROCK inhibitor) were purchased from Calbiochem. MK571 (CysLT1 inhibitor) was purchased from Cayman. AA861 (5-lipoxygenase inhibitor), Actinomycin D (Transcription inhibitor), Cycloheximide (Translation inhibitor), LY294002 (PI3K inhibitor), Mevastatin (HMG-CoA reductase inhibitor), MG132 (Proteasome inhibitor), SB203580 (p38 inhibitor), SB415286 (GSK-3 inhibitor), SP600125 (JNK inhibitor), U0126 (MEK inhibitor), and Epidermal Growth Factor (EGF) were purchased from Sigma. Herbimycin A (Hsp90 inhibitor) was purified from cultures of *Streptomyces* sp. in our own laboratory.

### Western Blotting

Following pre-treatment with either vehicle or inhibitors for 15 min, cells were treated with EGF for the indicated time. Cells were lysed in RIPA buffer (25 mM Hepes pH 7.8, 1.5% Triton X-100, 1% sodium deoxycholate, 0.1% SDS, 0.5 M NaCl, 5 mM EDTA, 50 mM NaF, 0.1 mM sodium vanadate, 1 mM PMSF, and 0.1 mg ml^−1^ leupeptin) at 4°C with sonication. The lysates were centrifuged and supernatants were recovered. After determining the concentration of the protein in each lysate, and boiling in an equal volume of loading buffer (150 mM Tris pH 6.8, 30% glycerol, 3% SDS, 15 mg ml^−1^ bromophenol dye, and 100 mM 2-mercaptoethanol), samples were then electrophoresed in a polyacrylamide gel. Proteins were transferred onto a PVDF membrane, and immunoblotted. Antibodies employed for immunoblotting were: anti-EGF Receptor antibody (#2232), anti-Akt antibody (#9272), anti-phospho-Akt (Thr308) antibody (#9275), anti-phospho-Akt (Ser473) antibody (#9271), anti-c-Jun antibody (#9165), anti-phospho-p38 antibody (#9211), anti-MAPK (ERK 1/2) antibody (#9102), anti-phospho-MAPK (ERK 1/2) antibody (#9101) (Cell Signaling); anti-c-Fos antibody (sc-7202), anti-p38 antibody (sc-7972), anti-p21 antibody (sc-397) (Santa Cruz Biotechnology); anti-phospho-tyrosine antibody (05-321) (Upstate); anti-phospho-c-Jun antibody (558036) (BD Pharmingen); anti-β-actin antibody (A5316) (Sigma).

### Statistical analysis

One-way ANOVA was used to determine significant differences in time series data sets. A Pearson product-moment correlation coefficient was used to confirm the reproducibility of the data. These statistical analyses were calculated by using the R (www.r-project.org/).

### Network deduction based on chemical biology

The signed directed network was deduced from a difference in protein phosphorylation or expression levels between mock-treated and inhibitor-treated data, according to the rule shown in **Figure 3**. This rule includes three simple assumptions that are commonly used in chemical biology: a) That there exists a positive regulation from molecule X to molecule Y when the level of molecule Y in the presence of both EGF and an inhibitor of X is more than 50% lower than in the EGF-treated condition; b) There exists a negative regulation from molecule X to molecule Y when the level of molecule Y in the presence of both EGF and inhibitor of X is more than 50% higher than in EGF-treated condition; c) There exists a negative regulation from molecule X to molecule Y when the level of molecule Y in the presence of inhibitor of X is higher than the half of the level in EGF-treated condition. The data processing which finally outputs pathway information consist of source node, target node, and regulatory type (positive or negative) was conducted by using R. This file is imported into Cytoscape software (www.cytoscape.org/), and signaling pathway maps were generated. The comparison of pathway topology was also performed using Cytoscape advanced network merge plugin.

## Supporting Information

Figure S1 **Representative immunoblot images presented in** **Figure 1** **.** (**a**) A431 cells, (**b**) EC109 cells, and (**c**) TT cells.
(PDF)

Figure S2 **Representative immunoblot images presented in** **Figure 2** **.** (**a**) A431 cells, (**b**) EC109 cells, and (**c**) TT cells.
(PDF)

Table S1 **The evaluation timeline of the effects of inhibitors.**
(DOCX)

Table S2 **Compound concentrations and targets of inhibition used in this study.**
(DOCX)
