# Correction: Chemical Genomic-Based Pathway Analyses for Epidermal Growth Factor-Mediated Signaling in Migrating Cancer Cells

**DOI:** 10.1371/journal.pone.0105243

**Published:** 2014-08-07

**Authors:** The PLOS ONE Staff

There are errors in Figure 2. The top label indicating EGF treatments, positive or negative, are inverse. Please see the corrected Figure 2 here.

**Figure 2:**
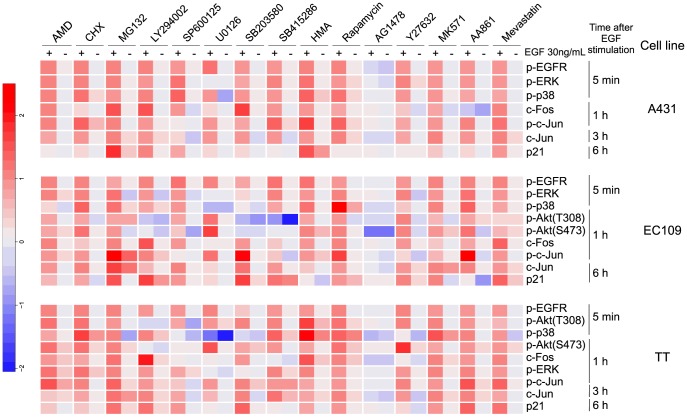
Effects of small molecule inhibitors on EGF-induced migration signaling in three cancer cell lines. Heat maps depict the effects of small molecule inhibitors on EGF-induced signaling in A431 cells (top), EC109 cells (middle), and TT cells (bottom). Data were normalized by centering non-treat condition as 0, and scaling EGF-treated condition to 1. All cell lines were treated with EGF after pre-treatment with inhibitors for 15 min. After the indicated time, the cells were collected and subjected to western blotting. AMD; Actinomycin D, CHX; Cycloheximide, HMA; Herbimycin A. The concentration of chemical inhibitors are listed in Table S2. Original immunoblot images are shown in Figure S2.
