# Supplementary figures and images for: Chemical Genomic-Based Pathway Analyses for Epidermal Growth Factor-Mediated Signaling in Migrating Cancer Cells
Source: PLoS One. 2014 May 12;9(5):e96776. doi: 10.1371/journal.pone.0096776 (PMC4018296; doi:10.1371/journal.pone.0096776)

Figure. S1

a

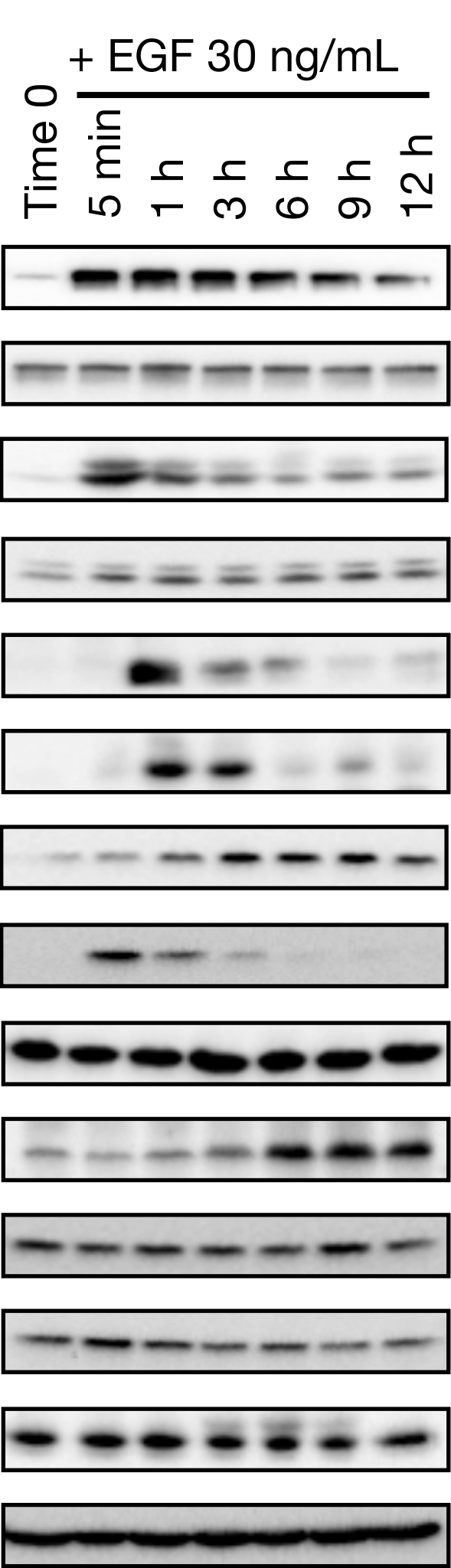

A431 cells

b

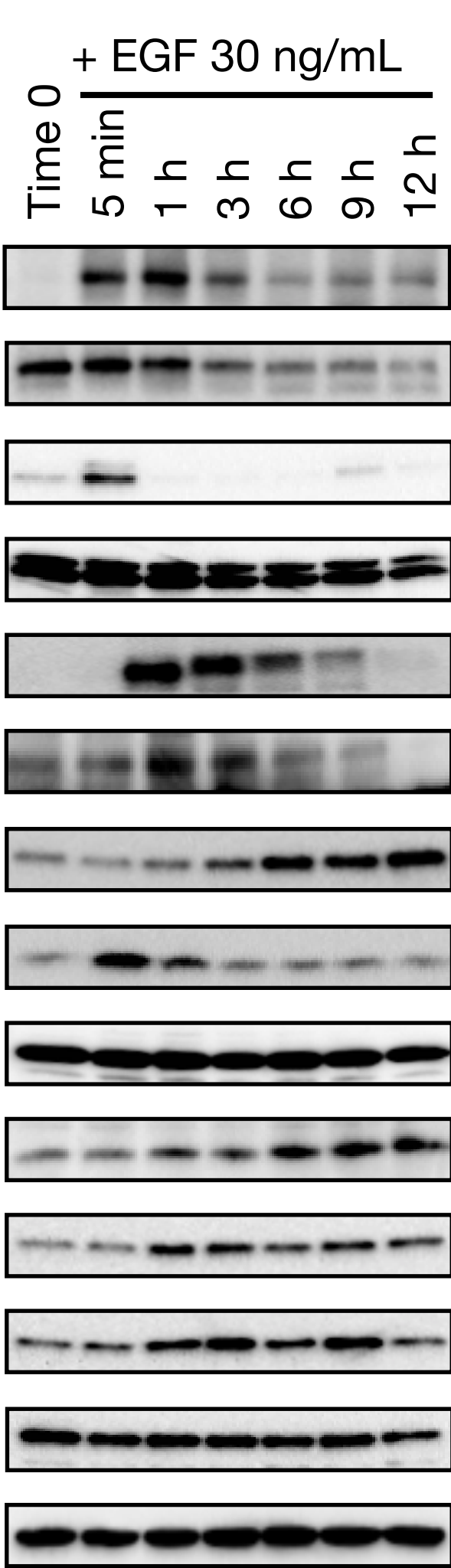

EC109 cells

c

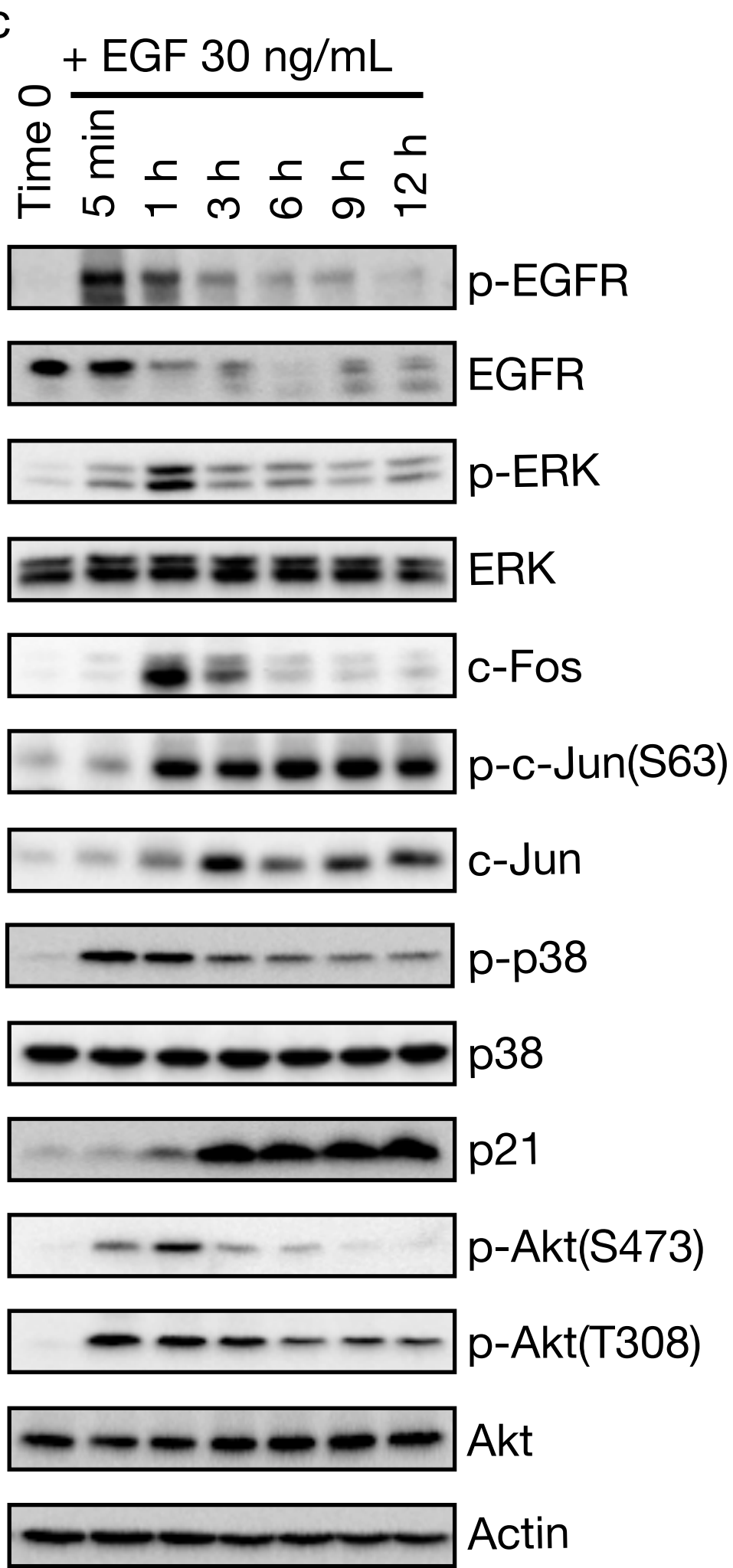

TT cells

Supplement: Figure S1 — Representative immunoblot images presented in Figure 1 . (a) A431 cells, (b) EC109 cells, and (c) TT cells. (PDF) [file pone.0096776.s001.pdf]

Figure. S2

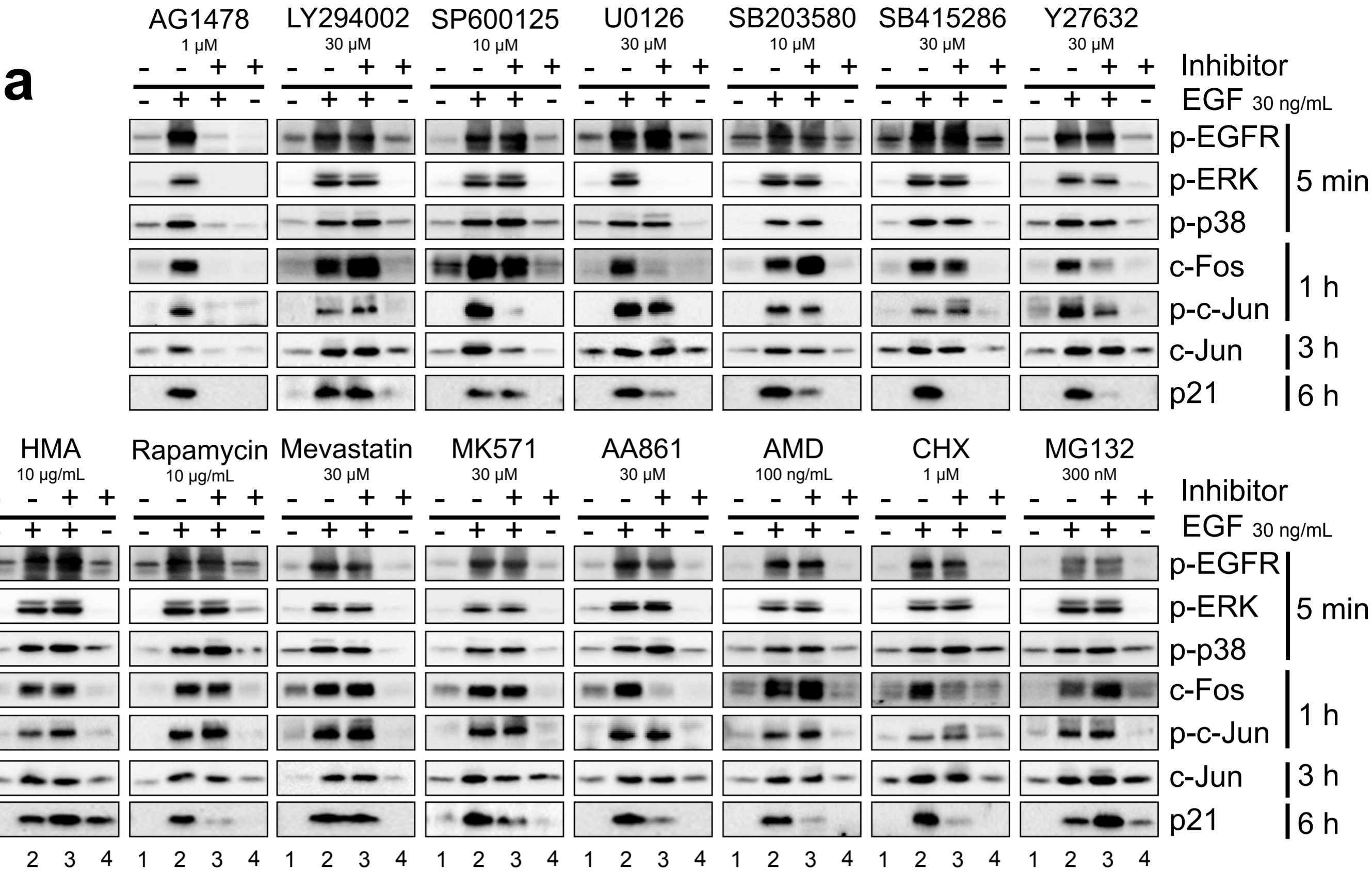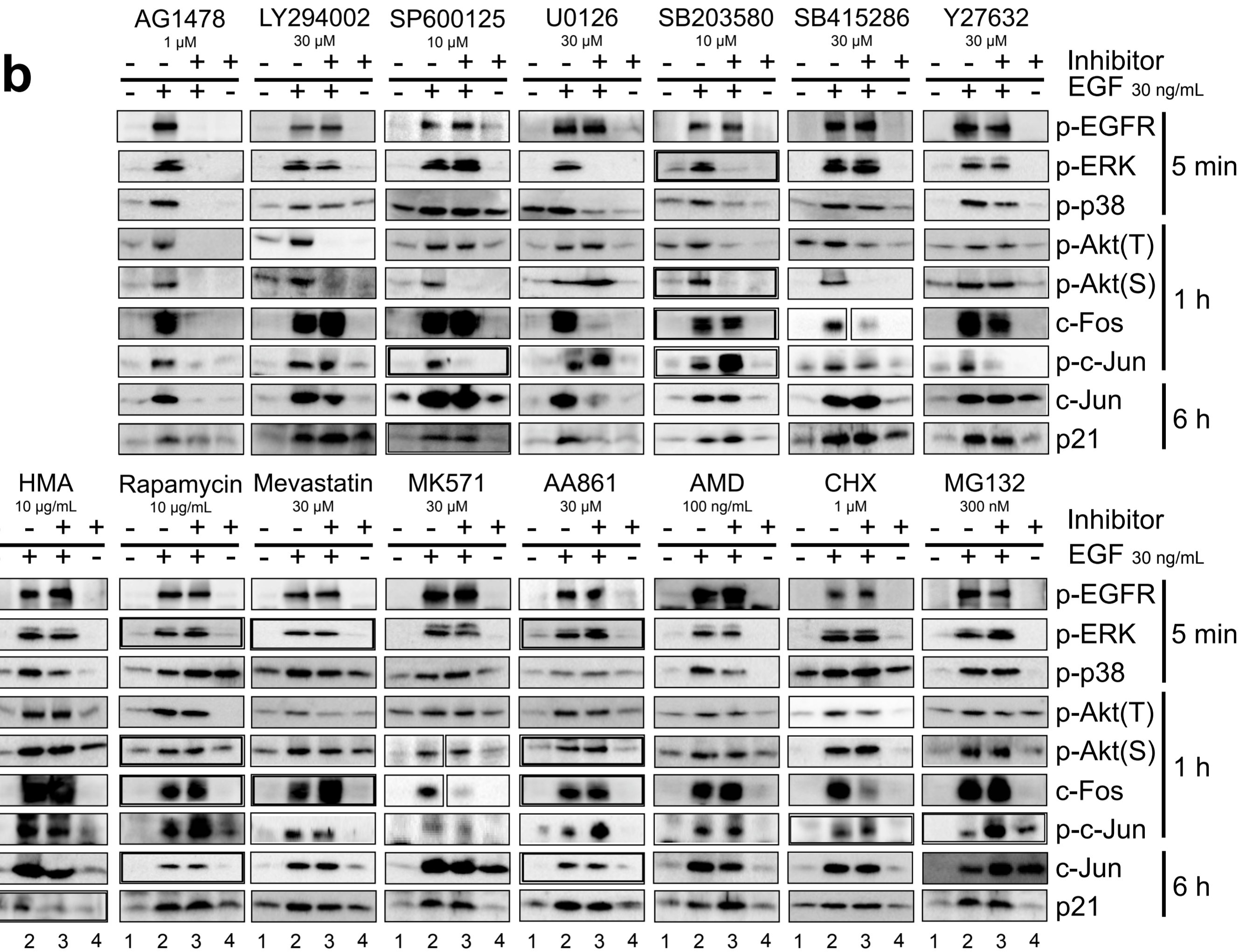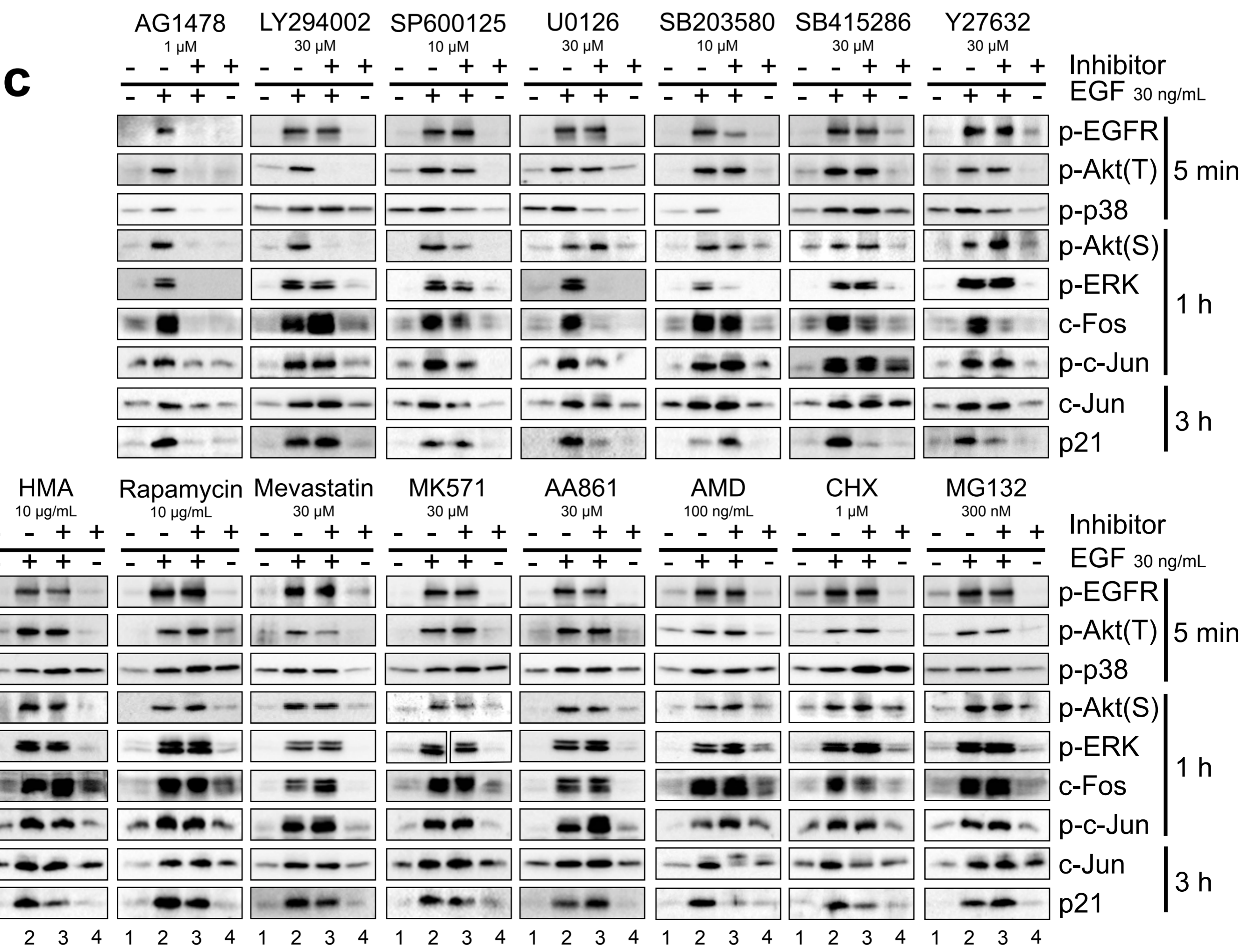

Supplement: Figure S2 — Representative immunoblot images presented in Figure 2 . (a) A431 cells, (b) EC109 cells, and (c) TT cells. (PDF) [file pone.0096776.s002.pdf]
