# Supplementary material for: Chemical Genomic-Based Pathway Analyses for Epidermal Growth Factor-Mediated Signaling in Migrating Cancer Cells
Source: PLoS One. 2014 May 12;9(5):e96776. doi: 10.1371/journal.pone.0096776 (PMC4018296; doi:10.1371/journal.pone.0096776)
Supplement: Table S1 — The evaluation timeline of the effects of inhibitors. (DOCX) [file pone.0096776.s003.docx]

Table S1. The evaluation timeline of the effects of inhibitors

| Proteins | Cell line | | |
| --- | --- | --- | --- |
|  | A431 | EC109 | TT |
| p-EGFR | 5 min | 5 min | 5 min |
| p-p38 | 5 min | 5 min | 5 min |
| p-Erk | 5 min | 5 min | 1 h |
| c-Fos | 1 h | 1 h | 1 h |
| p-Akt(T308) | – | 1 h | 5 min |
| p-Akt(S473) | – | 1 h | 5 min |
| p-c-Jun(S63) | 1 h | 1 h | 1 h |
| c-Jun | 3 h | 6 h | 3 h |
| p21 | 6 h | 6 h | 3 h |
