# Supplementary material for: Chemical Genomic-Based Pathway Analyses for Epidermal Growth Factor-Mediated Signaling in Migrating Cancer Cells
Source: PLoS One. 2014 May 12;9(5):e96776. doi: 10.1371/journal.pone.0096776 (PMC4018296; doi:10.1371/journal.pone.0096776)
Supplement: Table S2 — Compound concentrations and targets of inhibition used in this study. (DOCX) [file pone.0096776.s004.docx]

Table S2. Compound concentrations and targets of inhibition used in this study

| Compound name | Concentration | Target / Mode of action |
| --- | --- | --- |
| AA861 | 30 μM | 5-Lipoxygenase (5-LO) |
| actinomycin D | 100 ng ml^-1^ | Transcription |
| AG1478 | 1 μM | EGFR |
| cycloheximide | 1 μM | Translation |
| Herbimycin A | 10 μg ml^-1^ | Hsp90 |
| LY294002 | 30 μM | PI3K |
| Mevastatin | 30 μM | HMG-CoA reductase (HMG-CoA) |
| MG132 | 300 nM | Proteasome |
| MK571 | 30 μM | CysLT1 |
| Rapamycin | 10 μg ml^-1^ | mTOR |
| SB203580 | 30 μM | p38 |
| SB415286 | 30 μM | GSK-3 |
| SP600125 | 10 μM | JNK |
| U0126 | 30 μM | MEK |
| Y27632 | 30 μM | ROCK |
